# Diagnosis and Management of Cerebral Venous Thrombosis Due to Polycythemia Vera and Genetic Thrombophilia: Case Report and Literature Review

**DOI:** 10.3390/life13051074

**Published:** 2023-04-24

**Authors:** Dragos Catalin Jianu, Silviana Nina Jianu, Nicoleta Iacob, Traian Flavius Dan, Georgiana Munteanu, Anca Elena Gogu, Raphael Sadik, Andrei Gheorghe Marius Motoc, Any Axelerad, Carmen Adella Sirbu, Ligia Petrica, Ioana Ionita

**Affiliations:** 1 First Division of Neurology, Department of Neurosciences VIII, “Victor Babes” University of Medicine and Pharmacy, E. Murgu Sq., No. 2, 300041 Timisoara, Romania; jianu.dragos@umft.ro (D.C.J.); munteanu.georgiana@umft.ro (G.M.); anca.gogu@umft.ro (A.E.G.); 2 Centre for Cognitive Research in Neuropsychiatric Pathology (NeuroPsy-Cog), Department of Neurosciences VIII, “Victor Babes” University of Medicine and Pharmacy, 156 L. Rebreanu Ave., 300736 Timisoara, Romania; amotoc@umft.ro (A.G.M.M.); sircar13@yahoo.com (C.A.S.); petrica.ligia@umft.ro (L.P.); 3 First Department of Neurology, “Pius Brînzeu” Emergency County Hospital, 156 L. Rebreanu Ave., 300736 Timisoara, Romania; 4 Centre for Molecular Research in Nephrology and Vascular Disease, Faculty of Medicine, “Victor Babes” University of Medicine and Pharmacy, 156 L. Rebreanu Ave., 300736 Timisoara, Romania; 5 Department of Ophthalmology, “Dr. Victor Popescu” Military Emergency Hospital, 7 G. Lazar Ave., 300080 Timisoara, Romania; silvianajianu@yahoo.com; 6 Department of Multidetector Computed Tomography and Magnetic Resonance Imaging, Neuromed Diagnostic Imaging Centre, 300218 Timisoara, Romania; nicoiacob@yahoo.co.uk; 7 Department of Geriatrics-Rehabilitation, Riviera-Chablis Hospital, 3 Prairie Ave., 1800 Vevey, Switzerland; raphaelsadik@hopitalrivierachablais.ch; 8 Department of Anatomy and Embryology, “Victor Babes” University of Medicine and Pharmacy, E. Murgu Sq., No. 2, 300041 Timisoara, Romania; 9 Department of Neurology, General Medicine Faculty, Ovidius University, 900470 Constanta, Romania; 10 Department of Neurology, Central Military Emergency University Hospital, Clinical Neuroscience Department, “Carol Davila” University of Medicine and Pharmacy, 020021 Bucharest, Romania; 11 Division of Nephrology, Department of Internal Medicine II, “Victor Babes” University of Medicine and Pharmacy, E. Murgu Sq., No. 2, 300041 Timisoara, Romania; 12 Division of Hematology, Department V, “Victor Babes” University of Medicine and Pharmacy, E. Murgu Sq., No. 2, 300041 Timisoara, Romania; ionita.ioana@umft.ro; 13 Multidisciplinary Research Center for Malignant Hemopathies (CMCHM), “Victor Babes” University of Medicine and Pharmacy, E. Murgu Sq., No. 2, 300041 Timisoara, Romania

**Keywords:** cerebral venous thrombosis (CVT), polycythemia vera (PV), inherited (genetic) thrombophilia, intracranial hypertension syndrome, lateral sinus thrombosis, contrast enhanced three-dimensional T1 magnetization-prepared rapid acquisition gradient-echo (3D T1-MPRAGE) imaging

## Abstract

(1) Background: Cerebral venous and dural sinus thrombosis (CVT) rarely appears in the adult population. It is difficult to diagnosis because of its variable clinical presentation and the overlapping signal intensities of thrombosis and venous flow on conventional MR images and MR venograms. (2) Case presentation: A 41-year-old male patient presented with an acute isolated intracranial hypertension syndrome. The diagnosis of acute thrombosis of the left lateral sinus (both transverse and sigmoid portions), the torcular Herophili, and the bulb of the left internal jugular vein was established by neuroimaging data from head-computed tomography, magnetic resonance imaging (including Contrast-enhanced 3D T1-MPRAGE sequence), and magnetic resonance venography (2D-TOF MR venography). We detected different risk factors (polycythemia vera-PV with *JAK2 V617F* mutation and inherited low-risk thrombophilia). He was successfully treated with low-molecular-weight heparin, followed by oral anticoagulation. (3) Conclusions: In the case of our patient, polycythemia vera represented a predisposing risk factor for CVT, and the identification of *JAK2 V617F* mutation was mandatory for the etiology of the disease. Contrast-enhanced 3D T1-MPRAGE sequence proved superior to 2D-TOF MR venography and to conventional SE MR imaging in the diagnosis of acute intracranial dural sinus thrombosis.

## 1. Introduction

Cerebral venous thrombosis (CVT), including thrombosis of cerebral veins and intracranial dural sinuses, represents an underdiagnosed and less common cause of stroke (0.5–1% of all strokes in adults), but it is much more frequent than previously assumed [1,2,3,4,5].

In adults, CVT has a higher frequency among cases with inherited thrombophilia, mostly women (due to oral contraceptives, pregnancy, postpartum, or post-abortion) and patients with malignancy or infections [6,7,8,9,10,11,12].

Different Philadelphia-negative myeloproliferative neoplasms (MPNs) (including Polycythemia Vera–PV, essential thrombocythemia, and primary myelofibrosis) have an increased risk of venous thrombosis. However, previous studies indicate that CVT is rarely associated with MPNs (especially PV) [6,13].

Polycythemia vera (PV) is a *BCR::ABL1* negative, chronic MPN characterized by the proliferation and accumulation primarily of erythroid mass due to an abnormal clone of hematopoietic stem cells. This uncontrolled proliferation leads to an increase in hemoglobin (Hb) and hematocrit (HCT) levels and can be associated with an augmentation in the production of myeloid leukocyte cells and megakaryocytes [14].

The Janus kinase 2 V617F *(JAK2V617F)* mutation led to the diagnosis of PV, which rarely determines CVT [15].

This report describes an extremely rare case of thrombosis of multiple intracranial dural sinuses due to PV and genetic thrombophilia in an adult. The thrombosis affected the left lateral sinus (LS) (both transverse and sigmoid portions), the torcular Herophili, and the bulb of the left internal jugular vein (IJV). The patient clinically developed an isolated intracranial hypertension syndrome. He presented a history of PV with *JAK2V617F* mutation and a genetic low-risk thrombophilia (MTHFR A1298C heterozygote, Factor XIII homozygote, PAI 1 4G/5G heterozygote mutation). The clinical, neuroimaging, laboratory features, treatment, and the short outcome of our patient with CVT were presented and compared with those described in the literature.

## 2. Case Presentation

A 41-year-old male patient initially came to our Hematology Department in 2018, reporting mild asthenia and fatigue. He presented a history of grade II essential hypertension, right sub-segmental pulmonary thromboembolism (2017), venous thrombosis of the left lower limb (affecting popliteal and posterior tibial vein), and inherited low-risk thrombophilia (MTHFR A1298C heterozygote, Factor XIII homozygote, PAI 1 4G/5G heterozygote mutation).

During the clinical examination, plethoric facies, aquagenic pruritus, and erythromelalgia were noted. Physical examination revealed splenomegaly: spleen was palpable 1 cm below the rib cage. The neurological exam was also normal.

### 2.1. Laboratory Results

A complete blood count (CBC) noted leucocytosis: high white blood cell count (15.62 × 10^9^/L (normal range, 3.5–9.5 × 10^9^/L)), neutrophilia, thrombocytosis (362 × 10^9^/L (normal range, 135–350 × 10^9^/L)), increased red cell count (7.31 × 10^12^/L (normal range 4.35–5.65 × 10^12^/L)), a raised haemoglobin (Hb 185 g/L (normal range 132–166 g/L, grams per liter)), and increased hematocrit level (HCT 55.5% (normal range, 38.3–48.6%)).

Abdominal ultrasonography noted splenomegaly (with a maximum spleen length of 14.7 cm; normal values between 8.9 to 11.3 cm).

Bone marrow biopsy revealed age-adjusted hyper-cellularity with trilineage growth, including prominent erythroid, granulocytic, and megakaryocytic proliferation with pleomorphic, mature megakaryocytes, which suggested a histological aspect compatible with PV. The bone marrow biopsies performed in November 2017 and January 2022 (histopathological and immunohistochemistry exam) showed results of histopathological aspect compatible with PV. Additionally, the molecular biology from peripheral blood (genomic DNA isolation, ARMS-PCR amplification) revealed the presence of *JAK2 V617F* mutation homozygote (50% mutant clone); he also presented a subnormal serum erythropoietin level of 2.5 mU/mL (normal range, 3.1–16.5 milliunits per milliliter (mU/mL)).

Electrocardiography and cardiac ultrasonography showed the presence of sinus tachycardia, mitral valve prolapse, and low-grade mitral insufficiency.

Correlating the anamnestic, clinical, and laboratory data, the diagnosis of MPN PV with positive *JAK2V617F* mutation was established. In addition, based on personal thrombotic antecedents, the patient was included in the high-risk class. Risk-adapted therapy was considered. The treatment followed consisted of Alpha interferon (Intron A) (3 × 3 million UI/week), associated with periodic venesections, antiplatelet medication (Aspirin 75 mg/day, continuous), anticoagulant medication (Dabigatran etexilate 150 × 2/day, continuous), and specific cardiovascular treatment (Ramipril 5 mg/day, continuous).

In January 2022, the patient was readmitted to the Hematology department for reevaluation. Laboratory data revealed leukocytosis with neutrophilia, increased Hb and HCT levels, and thrombocytosis. The coagulation parameter measures noted an activated partial prothrombin time (APTT) of 73.6 s; thrombin officially standardized ratio (INR) of 1.07; prothrombin time (PT) of 13.5 s; fibrinogen content of 291 mg/dL; and quantitative D-dimer level of 138 ng/mL. No other relevant abnormalities were found.

Abdominal ultrasonography was performed in which splenomegaly (spleen = 14/5.5 cm) was detected. Bone marrow biopsy was reassessed and described a histopathological appearance compatible with PV. Based on the inadequate disease response described through splenomegaly presence, bone marrow alteration, progressive erythrocytosis, thrombocytosis, and leukocytosis, FDA-approved Ropeginterferon alfa-2b (100 µg) treatment was initiated in March 2022. We raised the dose every two weeks. The hematological parameters stabilized at 250 µg. We maintained this dose. Treatment was well tolerated without any presence of significant adverse effects.

In September 2022, the patient came to the emergency department (ED) of our hospital with acute onset (12 h) of severe (8/10) headache, initially associated with vomiting. The headache was generalized, augmenting gradually, and becoming permanent.

Upon physical examination, the patient presented redness of the skin, tachycardia, and moderate hypertension (HR = 92 bpm, BP = 155/100 mmHg), without fever.

Neurological examination was unremarkable.

Direct ophthalmoscopy and color fundus photography revealed bilateral papilledema but without visual complaints.

The ear-nose-throat (ENT) examination findings were normal.

His clinical diagnosis was established as isolated intracranial hypertension syndrome.

### 2.2. Neuroimaging Data

Unenhanced head CT realized in the ED of our hospital revealed hyperdensities along the left tentorium. We did not observe any parenchymal lesions or any air sinus abnormalities. He was admitted to our Department of Neurology with a probable diagnosis of CVT with a modified Rankin Scale (mRS) score of 1.

The patient underwent magnetic resonance imaging (MRI) combined with MR venography (MRV) with a 1.5-T MR unit in the first 12 h of admission in our Department of Neurology. The brain protocol included Sagittal T1W-weighted sequences; Axial diffusion-DWI sequences; Axial T2W; Axial fluid-attenuated inversion recovery-FLAIR; Axial T1W; post-contrast Axial T1W; and post-contrast 3DT1W.

We also used fast spoiled gradient-echo (FSPGR) and magnetization-prepared rapid acquisition gradient-echo (MPRAGE), both pulse sequence types belonging to the GRE family. We also used T2 star-weighted angiography (SWAN) sequence and advanced sequences, such as PROPELLER (Periodically Rotated Overlapping ParallEL Lines with Enhanced Reconstruction).

For post-contrast 3D T1W imaging, we used a 3D T1-weighted (3D-T1W) FSPGR sequence and a 3D T1-MPRAGE sequence.

**Figure 1:**
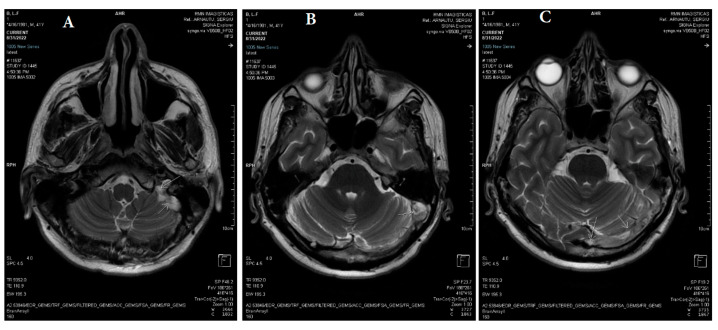
(**A**–**C**) Axial T2 PROPELLER: reduced flow in the left jugular bulb, left sigmoid sinus, left transverse sinus, and torcular Herophili (white arrows).

**Figure 2:**
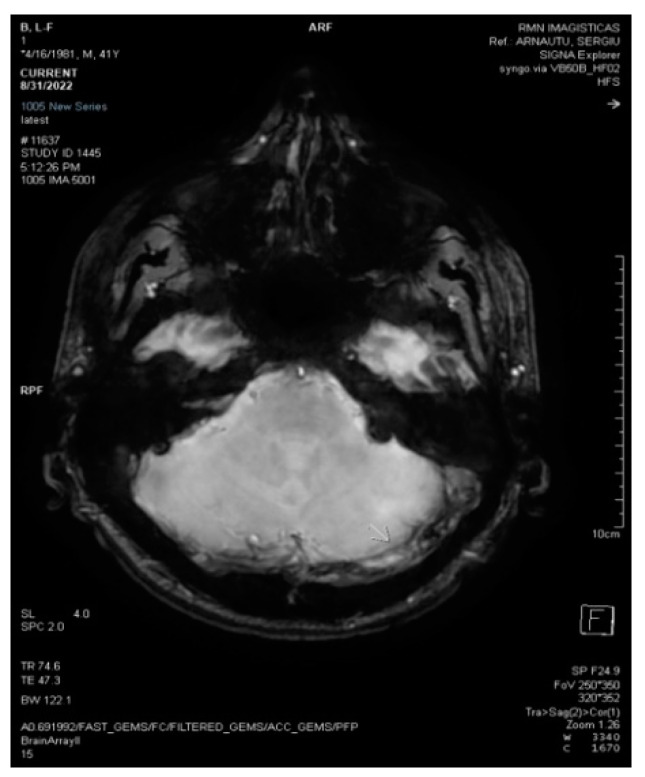
3D Axial SWAN shows acute thrombosis in the left transverse sinus (white arrow).

**Figure 3:**
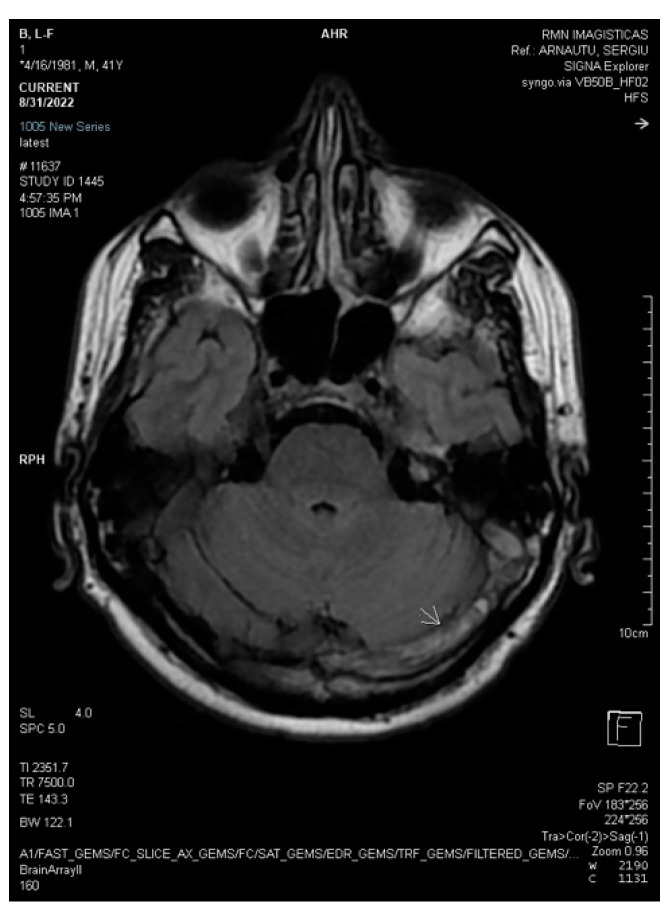
2D axial FLAIR shows reduced flow in the left transverse sinus (white arrow).

**Figure 4:**
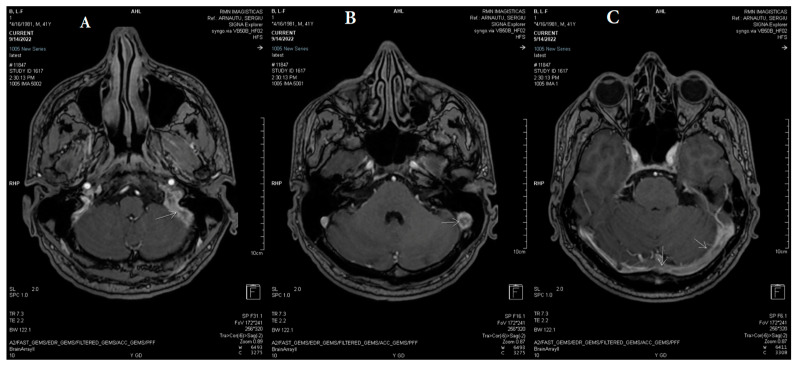
(**A**–**C**) Axial 3DT1 fast spoiled gradient-echo (FSPGR) post-contrast magnetic resonance demonstrates extensive filling defects throughout the dural sinuses (white arrows: left transverse sinus, left sigmoid sinus, left jugular bulb, and torcular Herophili).

The absence of parenchymal lesions was noted on the non-enhanced CT scan and the MRI (T1, T2, FLAIR, DWI, 3DT1-MPRAGE, 3DT1 FSPGR, and SWAN sequences). The diagnosis of acute thrombosis of the left LS (both transverse and sigmoid portions), the torcular Herophili, and the bulb of the left IJV was obtained based on the association of positive signs (definite spontaneous left LS hyperdensity on nonenhanced CT and hypointense signal of left LS, the torcular Herophili, and the bulb of left IJV on MRI sequences), and negative signs (filling defects with no visualization/absence of flow-related signal within the entire left LS, the torcular Herophili, and the bulb of the left IJV at MR venography (2D-TOF), denoting their occlusion) (Figure 1, Figure 2, Figure 3 and Figure 4).

### 2.3. Laboratory Tests

Laboratory tests revealed increased Hb and HCT levels, increased red cell count, leukocytosis with neutrophilia, and thrombocytosis. The coagulation parameter measures noted an activated partial prothrombin time (APTT) of 72 s, thrombin officially standardized ratio (INR) of 1.03, prothrombin time (PT) of 13.8 s, fibrinogen content of 284 mg/dL and quantitative D-dimer level of 142 ng/mL. No other relevant laboratory abnormalities were observed.

### 2.4. Treatment in the Acute Phase

Considering the CVT diagnosis, he immediately received low-molecular-weight heparin (LMWH) (Enoxaparin sodium-6000 IU twice a day).

Meanwhile, after three days of treatment with LMWH, the symptoms of headache and vomiting were gradually relieved, and he was discharged having recovered completely (MRS = 0).

### 2.5. Treatment after the Acute Phase

At discharge, due to his prothrombotic condition of PV with *JAK2V617F* mutation, and a genetic low-risk thrombophilia, we recommended oral anticoagulation (with dabigatran etexilate-150 mg twice daily) for an indefinite duration.

Neurological and imaging follow-ups were done at 30 days and three months after discharge, respectively.

We did not observe any recurrence (he did not present any other neurological symptoms/signs), deep vein thrombosis, or pulmonary embolism during this period of time.

**Figure 5:**
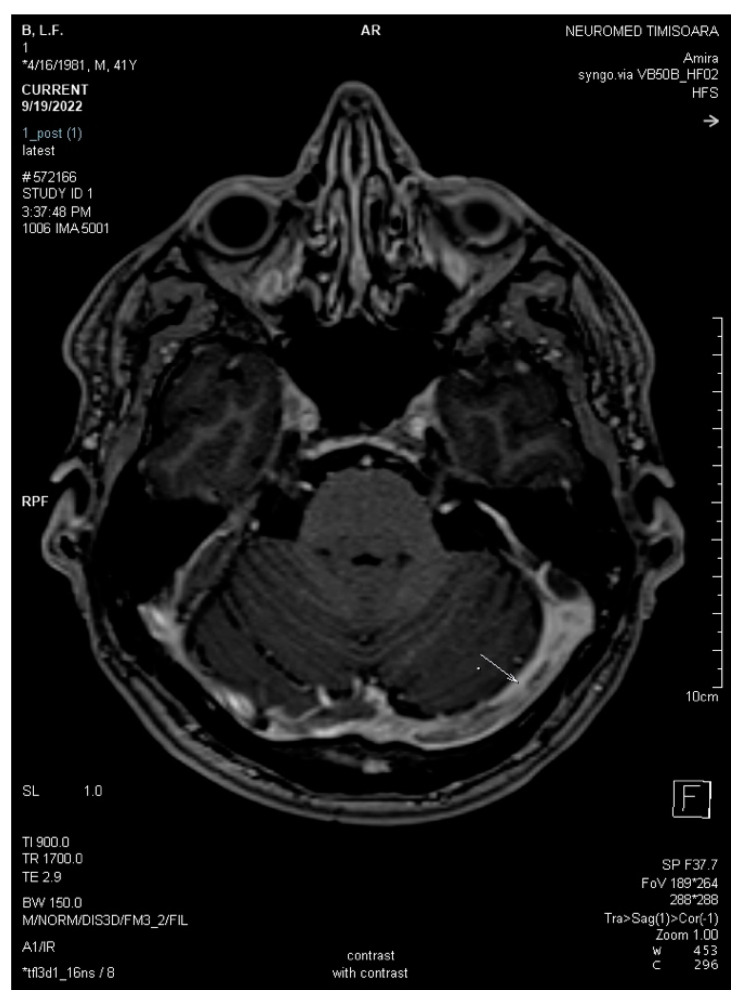
Axial 3DT1 MPRAGE post-contrast magnetic resonance after three weeks demonstrates unchanged filling defects throughout the dural sinuses (white arrows: left transverse sinus and torcular Herophili).

**Figure 6:**
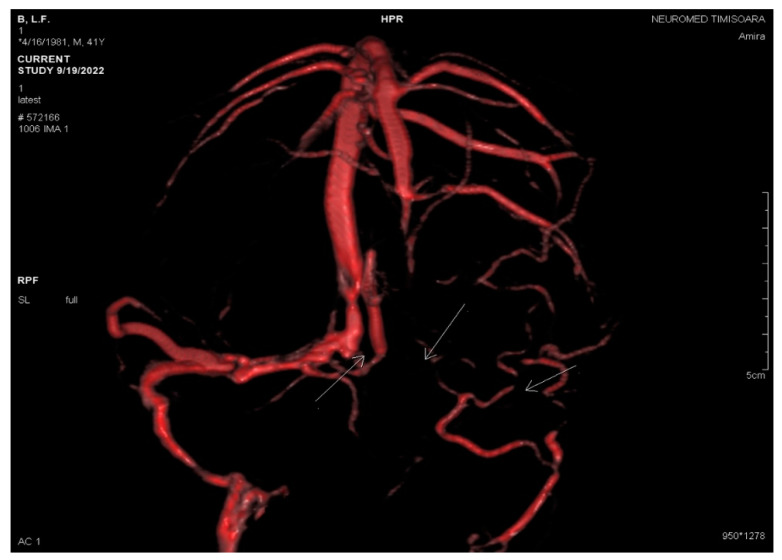
VRT reconstruction by PC 3DVENO sequence after three weeks: absent flow in the left jugular bulb, left sigmoid sinus, left transverse sinus, and torcular Herophili (white arrows).

**Figure 7:**
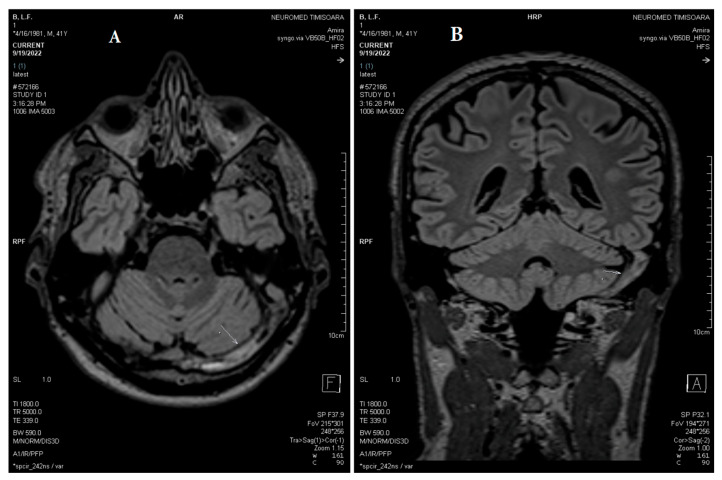
(**A**) Axial and (**B**) coronal MPR T2 spc_ir_dark_fl performed after three weeks shows unchanged reduced flow in the left jugular bulb, left sigmoid sinus, left transverse sinus, and torcular Herophili (white arrows).

The follow-up MRI/MRV demonstrated unchanged filling defects through the dural sinuses (Figure 5, Figure 6 and Figure 7).

Further testing was undertaken, so contrast-enhanced 3D T1-MPRAGE imaging was done on the patient. The images obtained more clearly revealed a hypointense signal of the left LS, the torcular Herophili, and the bulb of the left IJV (Figure 5).

## 3. Discussion

Clinical and imaging diagnosis of CVT is frequently difficult because of its nonspecific and variable clinical presentation and the overlapping signal intensities of thrombosis and venous flow on conventional MR images and MR venograms [10,11].

The clinical aspects of CVT are influenced by the following factors: site and number of occluded venous vessels, the functionality of collateral pathways, associated parenchymal lesions (vasogenic or cytotoxic edema, hemorrhage), age, gender, etiology, and interval from clinical onset to the beginning of the treatment [7,10,11]. A wide range of clinical presentations appears in patients with CVT. In adults, the most common clinical syndromes observed in combination or as isolated syndromes are intracranial hypertension, focal neurological deficits, seizures, and encephalopathy [1,2,3,16,17,18,19].

Isolated intracranial hypertension represents the most common clinical syndrome noted in CVT (nearly 40% of patients in the International Study on Cerebral Vein and Dural Sinus Thrombosis (ISCVT) cohort)) [6,7]. It is represented by headaches associated with vomiting, papilledema, visual symptoms, and sixth nerve palsy [20]. Headache is an extremely frequent neurological symptom of CVT (nearly 90% of patients in the -ISCVT cohort). It may be localized or diffused [20]. Papilledema is noted on funduscopy in 25–40% of CVT patients [6,7,8].

The patient in the current report presented with isolated intracranial hypertension with severe diffuse headache associated with vomiting and bilateral papilledema.

Two main pathophysiological mechanisms are implied in the genesis of CVT clinical spectrum: the first is represented by the diminution of cerebrospinal fluid (CSF) absorption, and the second by the progressive increase of venular and capillary pressure [12,21,22,23].

In our case, the isolated intracranial hypertension was produced by the complete thrombosis of different intracranial dural sinuses (left transverse sinus, left sigmoid sinus, left jugular bulb, and torcular Herophili), due to PV with *JAK2V617F* mutation, and to a genetic low-risk thrombophilia (MTHFR A1298C heterozygote, Factor XIII homozygote, PAI 1 4G/5G heterozygote mutation), with the consecutive diminution of CSF absorption [12,24,25].

According to the 2016 WHO classification, diagnostic criteria for PV are divided into two groups: major and minor [26]. On the one hand, the major criteria are defined by elevated Hb (>16.5 g/dL for men/>16.0 g/dL in women), HCT (>49% in men/>48% in women), or red cell mass level (>25% above mean normal predicted value); bone marrow pleomorphic changes such as panmyelosis and hypercellularity; and presence of *JAK2V617F* mutation, as in our case. On the other hand, the minor criterion for PV comprises a subnormal erythropoietin level. At least two major criteria plus the minor criterion are required for PV diagnosis [26].

PV is characterized by the clonal proliferation of hematopoietic stem cells, which determines the abnormal rise and accumulation of different circulating blood cells [27].

Consequently, PV produces hyperviscosity with stasis of blood (decreasing blood flow velocities) and eventually the appearance of thrombosis in different arterial and venous vessels.

The link between leukocytosis and thrombosis has been analyzed in different experimental studies based on the fact that in MPNs chronic and subclinical systemic inflammation presents an essential role in the pathogenesis of vascular events [28,29,30,31].

However, strong arguments supporting leukocytosis as an inflammatory biomarker potentially helping to differentiate prognostic categories in PV were still missing until recent studies noted that the neutrophil-to-lymphocyte ratio (NLR) represents an inexpensive and convenient predictor of venous thrombosis in PV [28,29,30,31].

Several groups of authors observed that arterial (AT) and venous (VT) thrombotic events are the most common complications in cases with PV and are the most important causes of morbidity and mortality [32,33,34].

In this context, these authors established that JAK2V617F VAF (variant allele frequency) >50% represented an independent strong predictor of VT (identifying patients with PV at high risk for VT), proving that AT and VT are different aspects which might require distinct management [32,33,34].

Thrombosis of intracranial dural sinuses affects CSF absorption, thus augmenting the intracranial pressure and producing the clinical syndrome of intracranial hypertension [5,35,36].

In addition, our patient had a *JAK2V617F* mutation, which represents an independent risk factor for thrombosis [15,37].

Inherited thrombophilias are the most important risk factors linked to CVT. Three mutations have been highly associated with CVT: factor V, Leiden; factor II, the pro-thrombin variant (PT 20210A); and the homozygosity for MTHFR C677T (severe genetic risk thrombophilia). These mutations were not relevant to our case because our patient presented only a low genetic risk of thrombophilia (MTHFR A1298C heterozygote, Factor XIII homozygote, PAI 1 4G/5G heterozygote mutation) [6,38,39].

Comprehensive knowledge of different anatomical variants of intracranial dural sinuses and cerebral veins is mandatory to identify CVT.

Each lateral sinus (LS) is located between the torcular Herophili and the IJV and contains two parts: the transverse segment (which lies on the attached border of the tentorium) and the sigmoid segment (which runs on the internal side of the mastoid process). The LSs collect venous blood from the posterior portions of the cerebral hemispheres, brainstem, and cerebellum. LSs also receive some of the diploic veins and some small veins from the middle ear, thus explaining their thrombosis in patients with mastoiditis or otitis media [1,2,3,4,5,6,12]. Frequently (in 50–80% of the cases), the two transverse sinuses are asymmetric, hypoplastic, or aplastic, transverse sinuses (usually the left one) being a relatively common variation (between 15% to 30% of all the patients) [1,2,3,4,5,6,12]. Therefore, the absence of a signal within a sinus, most commonly the left transverse sinus, may not always indicate thrombosis. It is usually suggestive of hypoplasia or aplasia, which was not present in our case [1,2,40,41,42].

Due to the great anatomic variability (in location, number, and anastomoses) of cortical veins and the posterior fossa veins, it is very difficult to identify their isolated occlusion [1,2,3,4,5,6]. In contrast to these two groups of veins, the deep cerebral veins are constant, and, in consequence, they are always identified at venography, thus any occlusion at their level being accurately detected, which was not our case [1,2,12,42].

Conventional MRI sequences, two-dimensional time-of-flight (2D-TOF) MR Venography, contrast-enhanced CT projection venography, and digital subtraction angiography (DSA) are common techniques in the diagnosis of dural sinus thrombosis [42].

On conventional MRI sequences, the dilated collaterals of the cerebral veins and dural sinuses and cerebral venous thrombosis are easily diagnosed in the subacute stage, but usually, they are not detected at the acute stage of CVT because the venous clot and the venous flow can produce overlapping signal intensities, as in our case [43].

With 2D-TOF MR Venography alone, it is very difficult to differentiate a hypoplastic or atretic dural sinus from its thrombosis. However, dural sinus thrombosis was presumed indirectly in our case by the absence of normal flow in the left lateral sinus since the thrombosis was usually isointense with the brain parenchyma [44]. A second pitfall of 2D-TOF MR Venography is represented by the signal loss of intracranial vessels, in which the direction of intracranial blood flow is within the imaging plane. This so-called saturation may resemble thrombotic occlusion [44,45,46].

According to different authors, high-resolution CT Venography has been considered superior to MR Venography (2DTOF or phase-contrast); however, we did not use this technique in our case because it presents a few disadvantages, including exposure to X-rays, the use of iodinated contrast material, poor delineation of skull base structures, and complex post-processing work [46,47].

Therefore, a noninvasive and more accurate diagnostic imagistic technique, the 3D contrast-enhanced T1 MPRAGE sequence, was used in our case for the acute diagnosis of intracranial dural sinus thrombosis [46,47].

According to different studies, this sequence is better than 2D-TOF MR Venography and conventional spin-echo (SE) imaging in the detection of both normal dural sinuses and cerebral venous anatomy and cerebral venous thrombosis, respectively, because it is not influenced by the angle between the cerebral vessel and the scan slab plane or flow velocities. For this reason, the MPRAGE sequence can excellently delineate cerebral veins and dural sinuses with good contrast and resolution between dural sinuses and neighboring cerebral lesions. The hypo-intense to intermediately-intense thrombosis was identified in our case because of excellent contrast of the thrombosis, the intensely enhanced sinus, and the adjacent brain parenchyma [48,49].

On the one hand, MPRAGE sequence makes possible the accurate diagnosis of an acute stage of CVT. Supplementary, the concomitant identification of dilated collateral veins and cerebral venous infarcts it is mandatory to predict prognosis with great confidence. Because of its superior ability to detect DSA, 3D contrast-enhanced MPRAGE represents an alternative as an efficient, noninvasive technique for the diagnosis and short-term follow-up of cases with acute CVT, as in our case [48,49].

On the other hand, this sequence may not be suitable in cases with chronic CVT due to organized and subsequently vascularized chronic venous clots because the enhanced thrombosis decreases the contrast with the normal intracranial dural sinus. Consequently, DSA and 2D-TOF MR Venography may be superior to 3D contrast-enhanced T1 MPRAGE in such cases [48].

The T2 star-weighted angiography (SWAN), or susceptibility-weighted angiography, is a high-resolution 3D multi-echo gradient echo (GRE) sequence that is more sensitive than GRE in the detection of cerebral hemorrhage and calcifications, which were not detected in our case. The SWAN sequence clearly identifies small blood vessels, microbleeds, and large vascular structures in the brain. SWAN also achieves larger images than GRE with a significantly higher contrast difference between the lesion and the healthy parenchyma, as in our case [50].

Head motion represents the main problem in magnetic resonance imaging (MRI). According to different authors, PROPELLER (Periodically Rotated Overlapping ParallEL Lines with Enhanced Reconstruction) MRI determines a sure means of quantifying and compensating for head motion, reducing motion artifact, and improving image quality compared with standard TSE sequences; thus intracranial pathology, including CVT, is better demonstrated with this technique, as in our case [51].

Therapeutic approaches for PV are predominantly targeting the clinical manifestation control increasing the quality of life by preventing the associated complications such as thrombotic events, secondary myelofibrosis, or acute leukemia transformation. Current treatment recommendations are adapted according to patient risk-adapted classification, so patients 60 years old or younger without prior history of thrombotic events are included as low-risk, while patients over 60 years old or with a history of thrombosis are considered high-risk, as the patient in our case was [52]. Cytoreductive therapy such as Hydroxyurea (HU), along with aspirin or phlebotomies, are generally accepted as first-line therapies in PV patients, but because of the frequency of HU intolerance, adverse effects, and disease progression, there was an increased need for modifying-disease agents. Interferons (INFs) and recombinant pegylated interferons have been studied as alternative therapeutic agents to HU. Recently, the usage of recombinant pegylated interferon alfa 2b in PV has been approved by the FDA with promising results, as in our case [53,54,55].

Given the confirmed CVT, anticoagulation was conducted in our case as the first-line therapy using initially body-weight-adjusted subcutaneous low-molecular-weight heparin, followed by oral anticoagulation (with dabigatran etexilate) [8,10].

Consistently, the clinical course of CVT was very good at three months. Several authors have suggested that recanalization of the occluded intracranial dural sinuses appears in 40–90% of CVT cases, the majority within the first four months of evolution, being reduced thereafter [6,7,8]. On the one hand, the cavernous sinus and the deep cerebral veins present a higher rate of recanalization; instead, the lowest rates were noted in LS occlusion, as in our case. On the other hand, recanalization of the thrombosed intracranial dural sinus is not related to the outcome after CVT, as in our case [6,7,8].

## 4. Conclusions

In our case, PV represented a predisposing factor for CVT, and the *JAK2V617F* mutation was helpful for diagnosis. Contrast-enhanced 3D T1-MPRAGE was superior to 2D-TOF MR venography and conventional SE MR imaging in the diagnosis of acute intracranial dural sinus thrombosis.

## Data Availability

First Department of Neurology, “Pius Brinzeu” Emergency County Hospital, Timisoara, Romania; Department of Multidetector Computed Tomography and Magnetic Resonance Imaging, Neuromed Diagnostic Imaging Centre, Timisoara, Romania.
